# Differential expression of an endogenous retroviral element [HERV-K(HML-6)] is associated with reduced survival in glioblastoma patients

**DOI:** 10.1038/s41598-022-10914-5

**Published:** 2022-04-27

**Authors:** Ashish H. Shah, Vaidya Govindarajan, Tara T. Doucet-O’Hare, Sarah Rivas, Leo Ampie, Catherine DeMarino, Yeshavanth Kumar Banasavadi-Siddegowda, Yong Zhang, Kory R. Johnson, Fahad Almsned, Mark R. Gilbert, John D. Heiss, Avindra Nath

**Affiliations:** 1 Surgical Neurology Branch, National Institutes of Health, National Institute of Neurological Disorders and Stroke (NINDS), Bethesda, MD USA; 2 Department of Neurosurgery, University of Miami School of Medicine, Miami, FL USA; 3 Center for Cancer Research (CCR), National Institutes of Health, National Cancer Institute (NCI), Neuro-Oncology Branch (NOB), Bethesda, MD USA; 4 Bioinformatics Section, National Institutes of Health, National Institute of Neurological Disorders and Stroke (NINDS), Bethesda, MD USA

**Keywords:** Cancer, CNS cancer

## Abstract

Comprising approximately 8% of our genome, Human Endogenous RetroViruses (HERVs) represent a class of germline retroviral infections that are regulated through epigenetic modifications. In cancer cells, which often have epigenetic dysregulation, HERVs have been implicated as potential oncogenic drivers. However, their role in gliomas is not known. Given the link between HERV expression in cancer cell lines and the distinct epigenetic dysregulation in gliomas, we utilized a tailored bioinformatic pipeline to characterize and validate the glioma retrotranscriptome and correlate HERV expression with locus-specific epigenetic modifications. We identified robust overexpression of multiple HERVs in our cell lines, including a retroviral transcript, *HML-6,* at 19q13.43b in glioblastoma cells. HERV expression inversely correlated with loci-specific DNA methylation. *HML-6* contains an intact open reading frame encoding a small envelope protein, ERVK3-1. Increased expression of ERVK3-1 in GBM patients is associated with a poor prognosis independent of IDH-mutational status. Our results suggest that not only is *HML-6* uniquely overexpressed in highly invasive cell lines and tissue samples, but also its gene product, ERVK3-1, may be associated with reduced survival in GBM patients. These results may have implications for both the tumor biology of GBM and the role of ERVK3-1 as a potential therapeutic target.

## Introduction

Human endogenous retroviruses (HERV) are ancestral remnants of previous germline retroviral infections and account for 8% of the human genome. HERV elements are identifiable by the presence of established retroviral genes: *gag* (core structural proteins), *pol* (viral replication enzymes including reverse transcriptase), *pro* (viral protease), *env* (envelope protein) and LTR (Long Terminal Repeats). In most differentiated cells, HERVs have lost their innate ability to form active viruses and replicate due to a combination of single nucleotide polymorphisms, coding deletions and mutations, but may nevertheless play roles in multiple physiological processes, including stem cell pluripotency, cell proliferation, and cell survival largely through their *cis*-regulatory elements found in their LTRs^1,2^.

However, several HERVs (mainly HERV-K, subtype HML-2) maintain variable degrees of transcriptional activity that are typically regulated by epigenetic mechanisms, including chromatin remodeling, DNA/histone methylation of transcription factors, and retroviral LTRs^3–6^. While these HERVs may also be expressed in healthy tissue, HERVs have been implicated in a number of disease processes, including autoimmune conditions and neurological disorders^7–10^. Furthermore, in cancer cells with known epigenetic dysregulation, HERVs have been implicated as potential oncogenic drivers^11,12^. For example, loss of CpG methylation in oncogenesis appears to impact HERV expression^13^. HERVs may contribute to cancer progression in several ways, including cis-activation of oncogenic promoters, direct protein formation or cell fusion^2,5,14^. HERV envelope proteins have been particularly implicated in multiple disease processes, including germ cell tumors and pancreatic cancer, suggesting that HERVs and their potential protein products may be associated with oncogenesis^15,16^.

In gliomas, HERV-K (HML-2) expression has been detected in specific tumor samples and cell lines through reverse transcriptase-qPCR; however, the extent and depth of HERV expression in gliomas has not been established to date^17,18^. Given the extent of HERV expression in many cancer cell lines and the known epigenetic dysregulation in gliomas, here, we conduct an exploratory analysis of three separate glioma cell lines (A172, H4, M059J) to characterize their retrotranscriptome, locus-specific gene expression, and differential methylation profiles. We sought to identify locoregional differential expression of endogenous retroviral elements, tumor suppressors and oncogenes in gliomas. Additionally, we investigated the link between HERV expression and locus-specific DNA methylation. Furthermore, we demonstrate that increased expression of one *HML-6* locus and its gene product, ERVK3-1, are associated with reduced survival in GBM patients.

## Results

### Differential HERV expression in glioma cell lines

**Figure 1:**
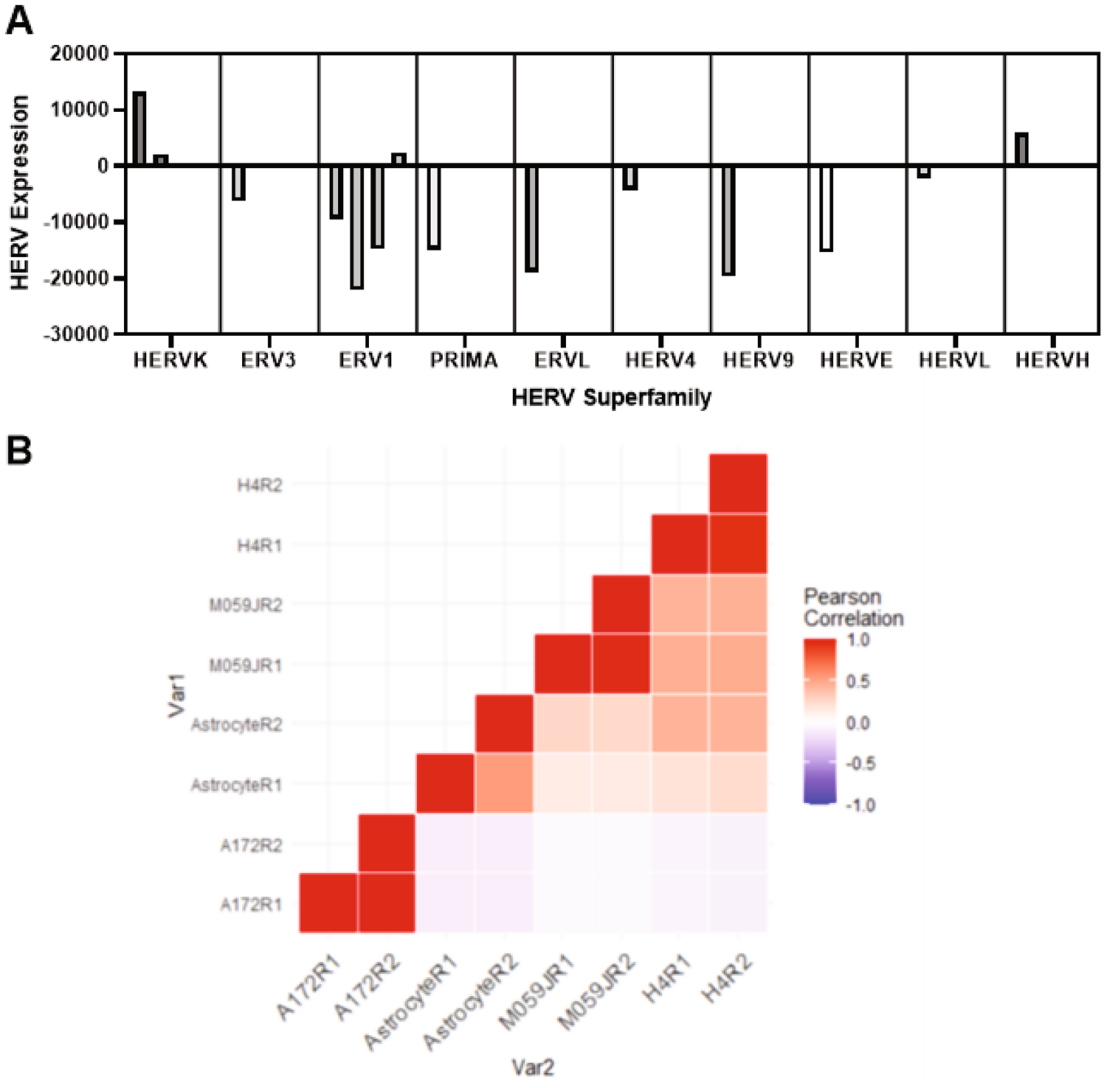
Among all investigated HERVs, certain HERV superfamilies demonstrated high degrees of dysregulation in the A172, M059J, and H4 cell lines in (**A**). (**B**) demonstrates the relative expression of highly dysregulated loci in each cell line and correlates HERV expression in each cell line Namely, A172 cell lines, which demonstrate the highest overall HERV expression, are most significantly correlated with each other, but have almost no association with other investigated cell lines.

Results of the HERV expression analysis were stratified by the ten most-dysregulated HERV families in each cell line. In the A172 cell line, the *HML-6* family had the highest expression (FC > 10,000, corrected *p* = 0.02), followed by ERV316A3 (*ERV3* superfamily, mean FC = 2838.564, corrected *p* = 0.009); while *MER61* (*ERV1* superfamily), *PABL-A* (*ERV1* superfamily, *PAB1XY*-Like sequences), and *PRIMA4 (PRIMA* superfamily) were all significantly under-expressed relative to astrocyte controls (FC = − 20,105.710, − 22,069.896, and − 14,892.596, respectively, corrected *p* = 0.019,0.028,0.016, respectively). The M059J cell line demonstrated significant underexpression of the *ERV316A3* (*ERV3* superfamily), *ERVLE* (*ERVL* superfamily), *HERV9*, *HERVE*, and *MER101* (*ERV1* superfamily) (corrected *p* = 0.033, 0.02, 0.009, 0.00058, 0.00016, respectively), and significant overexpression of *HERV4*, *HERVL*, *HML3* (*HERVK* superfamily), and *HERVH* families (corrected *p* = 0.0036, 0.012, 0.0037, 0.01). The H4 cell line similarly demonstrated significant underexpression of the *ERV316A3* (*ERV1*), *HERVE*, and *HERVL* (corrected *p* = 0.039, 0.025, 0.0019). The H4 cell line showed significant overexpression of *HERVIP10FH* (*ERV1*), *HML3* (*HERVK*), and *MER61* (*ERV1,*corrected *p* = 0.0008,0.0014,0.016). These findings are summarized in Fig. 1A and again in Supplementary Fig. 1. We performed a correlation and clustering analysis of HERV expression in our cell lines of interest, as shown in Fig. 1B. Our analysis demonstrated a high degree of association between A172 cell lines with respect to expression of all HERV loci, but minimal association with our other cell lines of interest.

**Figure 2:**
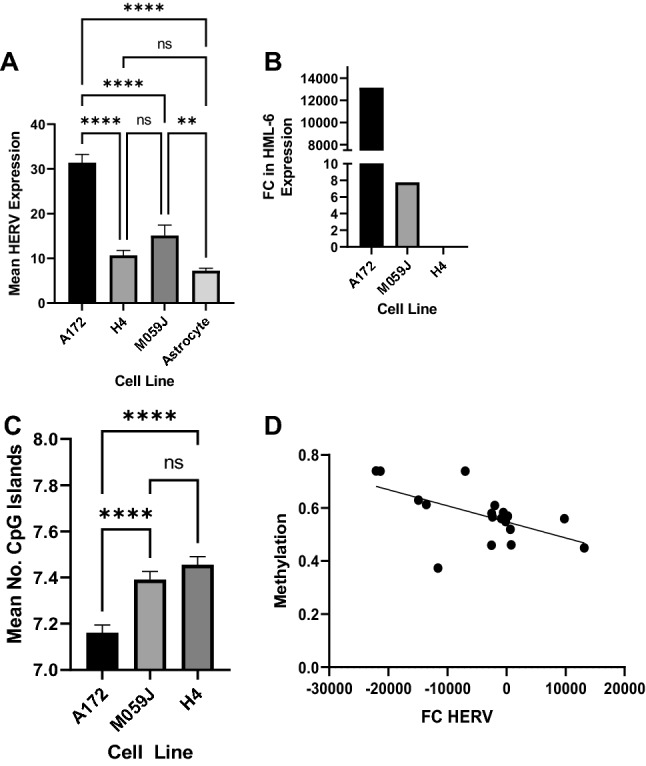
Mean HERV expression was highest in the A172 cell line (**A**), and HML6 is uniquely overexpressed in the A172 and M059J cell lines. However, HML6 expression was an order of magnitude higher in A172 relative to expression in M059J (**B**). Mean number of methylation sites, expressed as CpG islands, was lowest in the A172 cell line (**C**). Correspondingly, we found a significant inverse correlation between methylation (beta value) and log2 fold change (log2FC) in HERV expression across all investigated cell lines (**D**).

We analyzed differences in mean expression of overall counts among our cell lines of interest using one-way ANOVA under multiple comparison correction conditions. The results of this analysis demonstrated that the A172 cell line had significantly higher mean overall HERV expression relative to the M059J (mean difference, MD = 16.29, *p* < 0.0001) and H4 cell lines (MD = 20.74, *p* < 0.0001). There were no significant differences in mean HERV expression between the M059J cell line and H4 cell line (MD = 4.46, *p* = 0.22), though mean HERV expression in the M05J line was significantly higher relative to astrocyte controls (MD = 7.86, *p* = 0.004). Mean HERV expression in the H4 cell line was not significantly different from mean HERV expression in astrocyte controls (MD = 3.40, *p* = 0.46). A summary of these findings is shown in Fig. 2A. We also specifically analyzed HML-6 expression in our cell lines, which is displayed in Fig. 2B.

### Cellular methylation signatures

Analysis of CpG island methylation frequency (via ANOVA with multiple comparisons correction) in each cell line demonstrated that A172 cells had significantly lower mean number of CpG islands relative to M059J cells (MD = − 0.2, *p* < 0.0001) and H4 cells (MD = − 0.3, *p* < 0.0001). There were no significant differences in the mean number of CpG islands between M059J and H4 cell lines (*p* > 0.05). This data is summarized in Fig. 2C. Relative methylation (expressed as mean beta value) at the most differentially methylated loci in each cell line is presented in Supplementary Fig. 3.

We assessed the number of differentially methylated regions containing HERVs with a FC > 100 in each cell line. In the A172 cell line, 11 differentially methylated regions contained HERV loci, from an initial group of 100 loci. In the M059J cell line, 4 differentially methylated regions had HERV loci from an initial pool of 30 loci. In the H4 cell line, the 4 differentially methylated regions containing HERV loci were identified from an initial collection of 48 loci. When data from each cell line were pooled, our summative analysis demonstrated a significant inverse correlation between mean beta value and FC HERV expression (R = − 0.57, *p* = 0.01), as shown in Fig. 2D. Most notably, *HML-6* overexpression in A172 correlated with hypomethylation at the *HML-6* (19q43b) locus relative to astrocytes (*p* < 0.0001) as summarized in Supplementary Fig. 2.

### Validation of HML-6 expression

**Figure 3:**
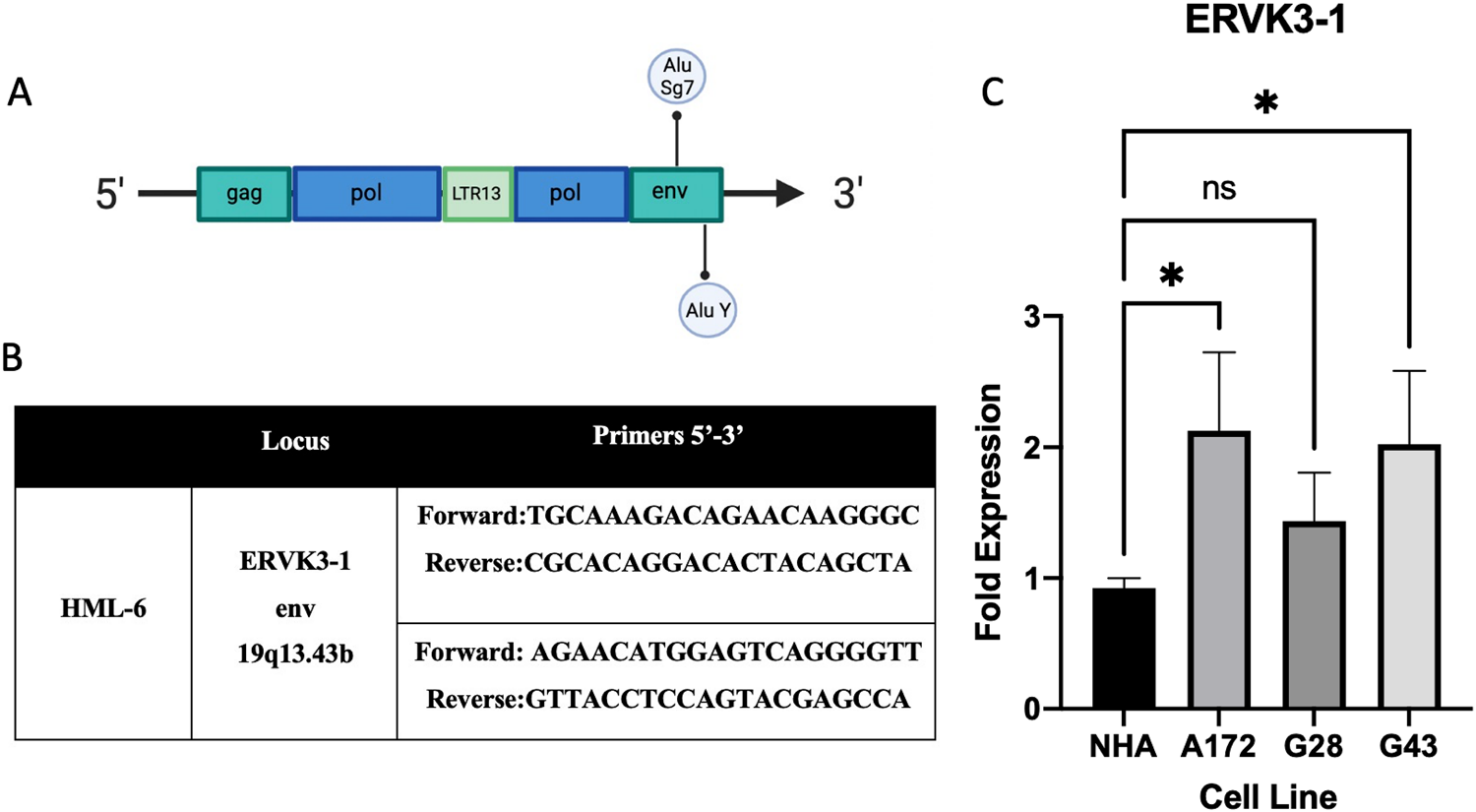
The HML-6 envelope transcript is overexpressed in GBM cell lines. (**A**) ERVK3-1 is located in the envelope region of the HML-6 provirus and encodes a small peptide (~ 140–190 amino acids). (**B**) Two sets of primers for the HML-6 provirus localize to the ERVK3-1 envelope region. (**C**) Quantitative Polymerase Chain Reaction (PCR) demonstrates significant overexpression of HML-6 in the adherent A172 glioblastoma cell line and patient-derived glioma neurosphere cultures (GBM 28 and GBM43). Data represents mean + SEM of triplicates and analyzed by multiple ANOVA. *p < 0.05.

**Figure 4:**
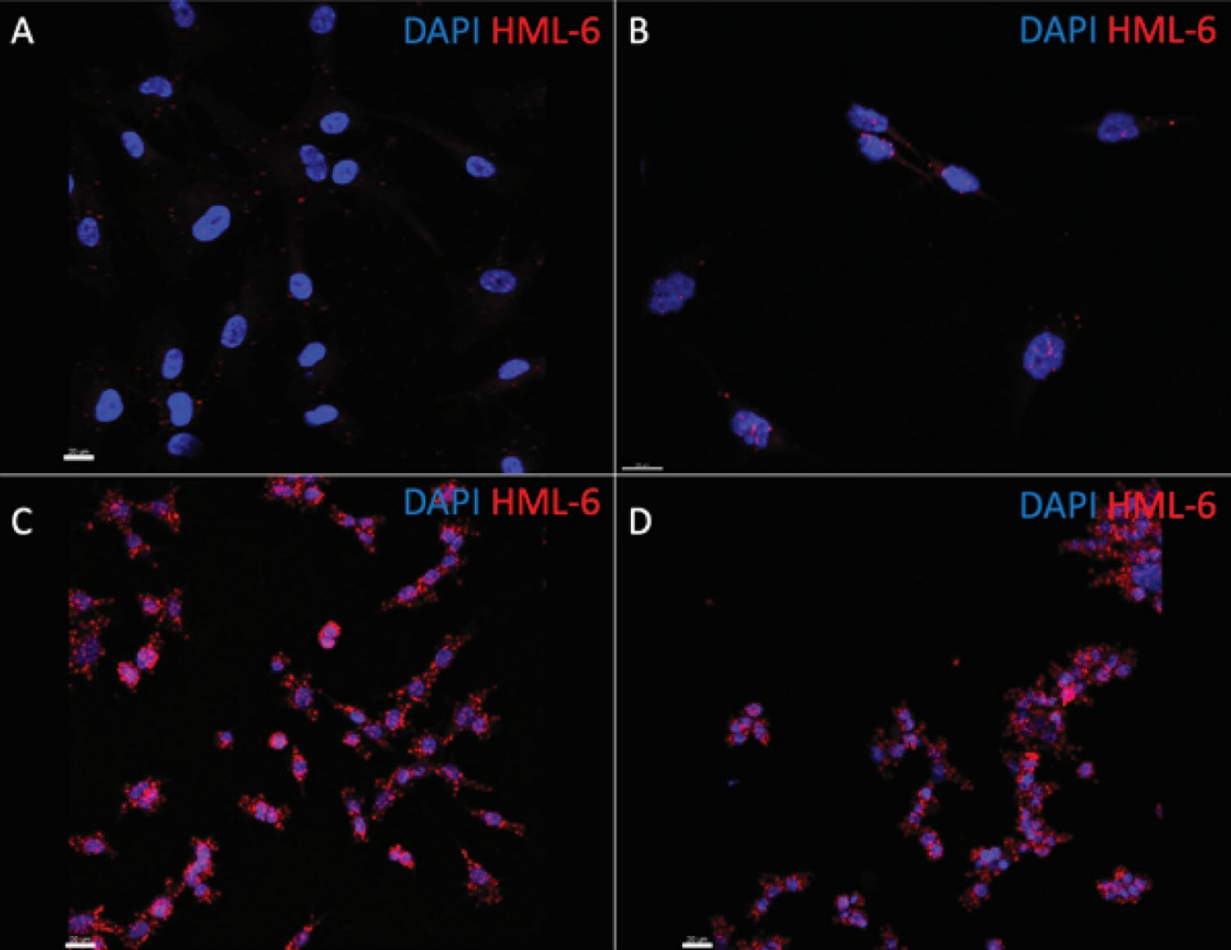
HML-6 is overexpressed in several glioma cell lines compared to astrocytes (**A**). RNA-in situ hybridization confirms presence of HML-6 provirus RNA in the nucleus (DAPI) and cytoplasm of glioblastoma cells. The adherent A172 glioblastoma cell lines (**B**) and neurosphere patient-derived glioblastoma cell lines, GBM28 (**C**) and GBM43 (**D**) have increased levels of HML-6 transcripts compared to astrocytes.

We validated our observations from the bioinformatic analysis where *HML-6* expression was highest in the A172 cell line by designing specific primers and probes that target *HML-6* for qPCR and RNA-in situ hybridization, respectively. Our primers and probes targeted the locus within the *env* region of the *HML-6* consensus sequence downstream to the LTR13 insertion within the *pol* region and overlapped the *env* region. This region is known to encode a small peptide called ERVK3-1. Our qPCR experiments based on the primers for the ERVK3-1 locus in the A172 adherent line and GBM neurosphere lines (GBM28 and GBM43) confirmed robust expression of the HML-6 locus (A172 > GBM43 > GBM28) and support our bioinformatics findings (Fig. 3). RNA in situ hybridization for *HML-6* was imaged by confocal microscopy and demonstrated marked over-expression of *HML-6* transcripts within all three GBM cell lines compared to astrocytes. (Fig. 4).

### Identification and functional analysis of differentially regulated genes

In addition to our identification of differentially methylated loci, we identified several similarly dysregulated genes (log2FC > 1.2). This analysis generated putative differentially expressed gene-HERV (DEG-HERV) pairs in the A172 and M059J cell lines, as shown in Supplementary Table 1, with a summary of DEGs and DEHERVs by cell line shown in Supplementary Table 1. Subsequently, DEGs were included in functional analyses in both cell lines. In the A172 cell line, DEGs were primarily associated with nucleotide synthesis. For example, CTP synthase 2 (*CTPS2*), the rate-limiting enzyme in cytosine nucleotide biosynthesis, was upregulated in our analysis and overlapped downregulated *PAB1XY*-like sequences (*PABL-A*, Xp22.2).

Similarly, several DEGs found in the A172 cell line were directly involved in cell division and growth. Most notably, the retroviral element, *ERV316A3*, and the RNA helicase *DDX25* were both over-expressed in the A172 cell line at the 11q24.211 locus. *DDX25* is a member of the DEAD-box protein family and is required for RNA processing/export, transcription, and translation initiation. A summary of overlapping DEGs and their functions are also shown in Supplementary Table 1.

### ERVK3-1 expression as a prognostic marker

**Figure 5:**
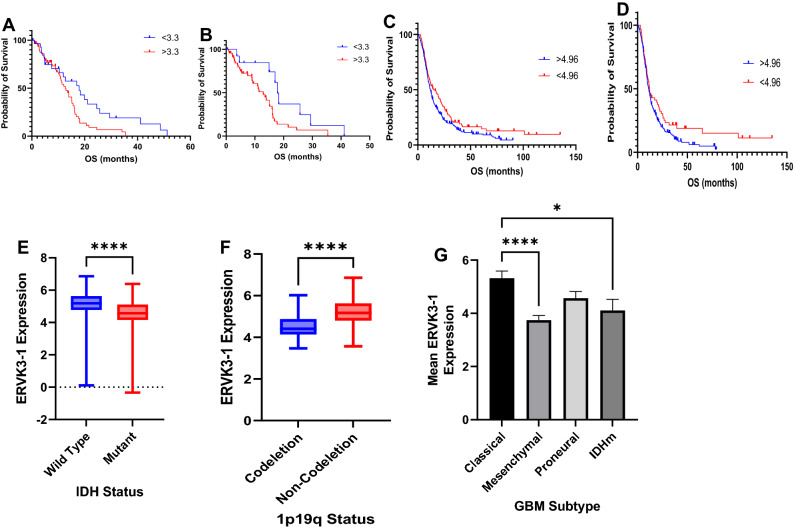
Integrated analyses from The Cancer Genome Atlas demonstrate that HML-6 envelope transcript, ERVK3-1, is a negative prognosticator in glioblastoma patients. (**A**) Across all samples, GBM patients with elevated ERVK3-1 expression had significantly lower median overall survival (17.9 months vs. 12.7 months, *p* = 0.018). (**B**) IDH-wild-type GBM patients with increased ERVK3-1 expression (> 3.3 FPKM) have a worse OS (17.9 vs. 12.9 months, *p* = 0.028). Analysis of CGGA data demonstrated significantly lower overall survival in patients with elevated ERVK3-1 expression, in all patients (median OS = 10 months vs. 19.4 months, *p* = 0.018) (**C)**. Among IDHwt patients alone, a similar trend was seen, though these differences were non-significant (median OS = 12.2 months vs. 11.3 months, *p* = 0.055) (**D**). In addition, patients with IDH mutant GBM had significantly lower ERVK3-1 expression relative to patients with IDHwt tumors (4.57 fpkm vs. 5.19 fpkm, *p* < 0.0001) (**E**), as did patients with 1p19q co-deletion (4.41 fpkm vs. 5.17 fpkm, *p* < 0.0001) (**F**). (**G**) Analysis of ERVK3-1 expression by GBM subtype revealed that the classical subtype has significantly higher ERVK3-1 expression than both mesenchymal glioblastomas and IDHm gliomas.

Analysis of TCGA and HPA data demonstrated that *IDH* wild-type (IDHwt) GBM patients with ERVK3-1 expression greater than 3.3 fragments per kilobase of transcript per million mapped reads (fpkm) had significantly lower median overall survival (OS) relative to patients with ERVK3-1 expression less than 3.3 fpkm (18.3 vs. 15.1 months, *p* = 0.039). This trend was preserved when including patients with IDH mutant (IDHm) GBM as well (17.9 months vs. 14.0 months, *p* = 0.0088). These findings are summarized in Fig. 5A,B, respectively. Similarly, analysis of CGGA data demonstrated significantly lower overall survival in patients with elevated ERVK3-1 expression, in all patients (median OS = 10 months vs. 19.4 months, *p* = 0.018), as shown in 5C. Among IDHwt patients alone, a similar trend was seen, though these differences were non-significant (median OS = 12.2 months vs. 11.3 months, *p* = 0.055, 5D).

In addition, patients with IDH mutant GBM had significantly lower ERVK3-1 expression relative to patients with IDHwt tumors (4.57 fpkm vs. 5.19 fpkm, *p* < 0.0001), as did patients with 1p19q co-deletion (4.41 fpkm vs. 5.17 fpkm, *p* < 0.0001). These findings are shown in Fig. 5E,F. When stratified based on GBM subtype, analysis of ERVK3-1 demonstrated that classical GBM samples had significantly higher ERVK3-1 expression relative to mesenchymal (5.32 fpkm versus 3.74 fpkm, corrected *p* < 0.0001) and IDHm GBM samples (5.32 fpkm versus 4.11 fpkm, *p* = 0.022). These findings are summarized in Fig. 5G.

## Discussion

Here, we performed the first comprehensive analyses of HERV expression and methylation signatures in established glioma cell lines. Our research of overall HERV expression in glioma cell lines has demonstrated differential HERV expression among commercially available glioma cell lines; A172 showed the highest mean HERV expression relative to M059J and the non-tumorigenic H4 cell lines.

Of note, no predominantly overarching HERV family was consistently expressed in these established cell lines potentially due to the significant heterogeneity. One possibility is that each glioma cell line is a distinct entity with its own characteristic endogenous retroviral and methylation signature. Alternatively, if larger sample sizes of glioma cell lines were to be analyzed, patterns of HERV expression might emerge that could help categorize the tumors based on HERV expression.

The highly proliferative A172 cell line demonstrated a marked overexpression of one specific HERV: *HML-6* at chromosome 19q13.43b. *HML-6* is considered part of the beta retrovirus-like viruses (Class II) and contains at least one open reading frame (ERVK3-1) and several pro-viral loci^12^. Previous studies using microarrays have suggested that extensive transcriptional activity of *HML-6* exists in a wide variety of normal tissues, including the brain^19^. However, increased expression of *HML-6* pro-viral loci has also been noted in several cancers, including breast cancer and melanoma^20,21^. Specifically, small antigenic peptides from the *HML-6* env gene (HERV-K-MEL) have been isolated from melanoma tissues, which are recognized by cytotoxic T-cell lymphocytes^22^. Similar studies have described low-level transcriptional activity of LTR retrotransposons in normal tissues, and subsequent clonal expansion of these retroelements in tumors^23^. Prior research has also demonstrated that unique HERVs may drive oncogenesis, and that clusters of phylogenetically distinct HERVs may be common among different cancers, as shown by Steiner et al. However, while the authors did note multiple members of the HERV-K family to be dysregulated in their analysis, *HML-6* was not described^24^.

The discovery of these cancer-specific epitopes suggested a role of *HML-6* in cancer, but its oncogenic potential has not been well-defined. Since *HML-6* was markedly overexpressed at one specific locus (19q13.43b) in the A172 cell line, we focused on validating our bioinformatics work on this locus. This locus also contains the open reading frame for *HML-6* protein (ERVK3-1, Entrez ID: 105,372,481), corresponding to its *env* gene. Our qPCR validated increased transcript expression in both the adherent A172 glioma cell line and neurosphere patient-derived glioma lines (GBM28 and GBM43) compared to normal human astrocyte cell lines. Due to this association, further investigation into the oncogenic potential of *HML-6* may be warranted. Currently, there are no commercially available antibodies to the putative *HML-6* env protein hence protein expression could not be confirmed.

Our analysis suggests an inverse relationship between HERV expression and DNA methylation at several differentially expressed HERV loci across all cell lines (*p* = 0.01). Previously, HERV expression has been inversely correlated to DNA methylation in several cancers^25–28^. In normal differentiated cells, transcriptional regulation of HERVs is tightly controlled by DNA methylation; however, in embryonic stem cells and some cancers, HERV expression also relies on other epigenetic modifications such as histone methylation (H3K9me3) and acetylation (H3K27Ac^4,29^. A recent retroelement specific array demonstrated DNA hypomethylation for several HERV superfamilies (HERV-K, HERV-H, HERV-W) in head and neck cancer patients compared to adjacent normal tissues^30^. Similar to our findings, loss of CpG island methylation has also been associated with increased HERV and LINE-1 element expression in ovarian cancer and embryonal cancer cell lines^31,32^. This relationship has been further defined in several cancers where exposure to demethylating agents (5-Azacytidine) induces expression of endogenous retroviral elements^25,33,34^.

The glioma CpG Island Methylator Phenotype (G-CIMP) comprises a unique subset of gliomas with a distinct epigenetic landscape. G-CIMP gliomas possess marked DNA methylation and upregulation of key histone methyltransferases and deacetylases that result in global transcriptional silencing, which may silence retroelement expression. As gliomas progress to higher grades, loss of the G-CIMP phenotype has been described, suggesting a potential link between HERV expression and glioma progression^35,36^. Nevertheless, the heterogenous epigenetic landscape of gliomas may complicate investigations of the role of epigenetic regulation of HERVs in gliomas. Given our collective findings, further studies into the role of epigenetic dysregulation of HERV elements in gliomas are warranted.

Our analysis of ERVK3-1 expression in clinical samples has demonstrated a potential survival detriment in patients with elevated ERVK3-1 expression, marking the first such analysis of a HERV transcript as a negative prognostic marker in GBM. Given the heterogeneity of GBMs, Our subgroup analysis confirmed heterogeneous expression of ERVK3-1 in several GBM subtypes with the highest expression in the classical GBM patients. The classical subtype accounts for approximately 25% of malignant gliomas, and is characteristically marked by Epidermal Growth Factor Receptor (EGFR) amplification/mutation. In concordance with our findings of overexpressed HML-6 expression in GBM cell lines, the two patient-derived neurosphere lines were both derived from patients harboring the classical GBM subtype. In addition, *IDH* mutant GBM patients had relatively lower ERVK3-1 expression; given that HERV expression is closely linked to epigenetic dysregulation, the high degree of methylation seen in IDHm GBM may explain this finding^3–6,37^. The results of our study have several implications in both the general tumor biology of GBM and in clinical applications as well. Primarily, we suggest that *HML-6* may be associated with GBM invasiveness, given its overexpression in our more invasive A172 cell line and patient-derived neurospheres. The potential role of *HML-6* as a driver of GBM oncogenesis may center on its unique gene product, ERVK3-1. While multiple *HML-6* sites are present in the genome, only the locus at 19q13.43b contains an intact reading frame for ERVK3-1. Our most salient future studies will focus on elucidating the mechanism underlying *HML-6* driven oncogenesis and the potential impact ERVK3-1 may have in this process. One potential role ERVK3-1 may have in oncogenesis is immunomodulation^21^. For example, HML-6 gene products have been previously implicated in immunomodulation, as demonstrated by Schiavetti et al. Their results demonstrated that the HERV-K-MEL envelope protein (located on chromosome 16) may be an epitope for cytotoxic lymphocytes (CTL) against melanoma. Thus, future studies investigating the relationship between ERVK3-1 expression and the tumor immunophenotype would provide critical insight in this regard.

Clinically, the impact of ERVK3-1 on patient survival from curated tissue samples is intriguing. While our work has formed the basis for ERVK3-1 to be considered a potential prognostic marker for GBM survival, development of a curated database with tumor samples sequenced for HERV and ERVK3-1 expression would be highly beneficial for future clinical application of our findings. In addition, obtaining data from paired primary and recurrent GBM samples would grant further insight into the role of HML-6/ERVK3-1 in tumor recurrence. Further analysis of the role of *HML-6* and ERVK3-1 with respect to tumor biology may present avenues for assessing ERVK3-1 as a drug target.

Our analysis aimed to validate previously described bioinformatic pipelines in established commercially available glioma cell lines with whole-cell RNA-seq data. Although we demonstrated several overexpressed loci, each cell line demonstrated heterogeneous HERV expression, suggesting the absence of one overarching HERV family. Therefore, it remains essential to analyze the retrotranscriptome of each glioma cell line separately. Additionally, several studies have suggested that adherent glioma cell lines do not accurately recapitulate the glioblastoma disease phenotype. Therefore, utilizing patient-derived glioma stem-like cells offers a potential option for uncovering novel HERV transcripts in gliomas.

Here, we confirmed the upregulation of *HML-6* in several glioma cell lines; however, we cannot make any conclusions on other glioma cell lines based on our analysis. In addition, RNAseq reads were mapped to the human genome as opposed to the cell line genomes. As these cell lines genomes fundamentally differ from the standard human genome, this may have similarly affected our analysis of HERVs in our cell lines of interest. Given the heterogeneity of gliomas, a tailored bioinformatic pipeline for each cell line may help define the retroviral landscape for each patient. Future investigations could focus on the mechanistic interactions between the DEG-HERV pairs to validate whether certain HERVs alter functional gene expression through antisense, or proto-oncogene activation.

Lastly, when running any systematic analyses for characterization of endogenous retroelement expression, a custom RNA-seq dataset is required. We typically recommend that RNA-seq datasets should be adequately prepared (ribosomal RNA depleted, non-poly-A tail) with sufficient reading depth (> 100 bp, ~ 50 million reads) to detect HERV sequences accurately. Although the TCGA dataset is not ideal for such analyses, we conducted a preliminary survival analysis to query the prognostic impact of *HML-6* in GBM patients. Future extensive bioinformatic studies with a tailored transcriptomic dataset of patient tumors will help clarify and validate the landscape of endogenous retroviruses in gliomas.

## Conclusion

In gliomas, HERV expression directly correlates with loss of DNA methylation at the corresponding HERV loci. Of differentially expressed HERVs, *HML-6* is overexpressed in a subset of highly invasive glioblastoma cell lines and patient-derived neurospheres. We have also demonstrated a potential survival detriment associated with elevated expression of the *HML-6* product, ERVK3-1. Further analysis of ERVK3-1 is necessary to elucidate its role in glioma oncogenesis and recurrence. Our results have demonstrated several implications for the role of *HML-6* and ERVK3-1 in the tumor biology of GBM as well as the potential clinical applications of future studies.

## Methods

### HERV expression of glioma cell lines from ENCODE datasets

Three glioma cell lines (A172 (53-year old male), M059J (33-year old male), H4 (37-year old male)) and control (human fetal astrocytes) were identified from whole-cell RNA-seq data from the ENCODE (ENCyclopedia Of DNA Elements) Consortium (encodeproject.org). Tier 1 sequence data was obtained from Sequence Read Archive (SRA) and aligned to the reference genome (hg38) using bowtie2. We utilized only paired-end ribosomal depleted samples from ENCODE with RNA fragments > 200 bp in our analyses. All cell lines originated from male patients; therefore, no gender-specific analysis was required. We ran the Telescope algorithm with default parameters on the resulting aligned BAM files using a previously published annotation of HERV locations (GTF) (https://github.com/mlbendall/telescope_annotation_db). When final counts from the TSV file were retrieved, our initial HERV data set contained 2367, 1374, and 1243 unique loci for HERV expression in A172, M059J, H4, respectively^38^.

### Study approval

Our research did not utilize human or animal subjects, and all clinical sample data was open-source and deidentified. Thus, ethical approval from an IACUC or IRB was not required.

### Differential expression of HERVs and nearby genes

To characterize HERV differential expression, we utilized a previously published custom ensemble package in R, DESeq2, to conduct noise-modeling, data filtering, and statistical analysis of mean locus-specific HERV expression in the cancer cell lines relative to astrocyte controls. Differentially expressed HERVs (DE-HERVs) were considered significant if their expression crossed a minimum threshold of corrected *p* < 0.05 (t-test with Benjamini–Hochberg correction) and absolute FC > 1.2. Differentially expressed HERVs were noted in 39.9% (945/2367), 4.8% (66/1374) and 9.8% (122/1243) of all the unique HERV loci in the A172, M059J, and H4 cell lines, respectively. Differential gene expression (DEG) in each cell line were analyzed for overlapping and neighboring genes at dysregulated HERV loci using the R packages dplyr and Genomic Ranges.

When a DEG (differentially expressed gene with log_2_FC > 1.2 and Benjamini–Hochberg corrected *p* < 0.05) and DE-HERV (differentially expressed HERV) coincided in differential expression (both HERV and gene were upregulated or downregulated, log_2_FC > 1.2, *p* < 0.05), a DEG-HERV pair was noted, as previously described^39^.Among our differentially expressed HERV loci with differential methylation, we determined whether there were interceding antisense RNA sequences that may also result in differential HERV expression using RepeatMasker (University of California Santa Barbara) on the opposing strand of interest. Proximity to the gene nearest to each HERV locus was calculated using the R package Genomic Ranges via the ‘DistanceToNearest’ function. KEGG.

### Cell line methylation analyses

**Figure 6:**
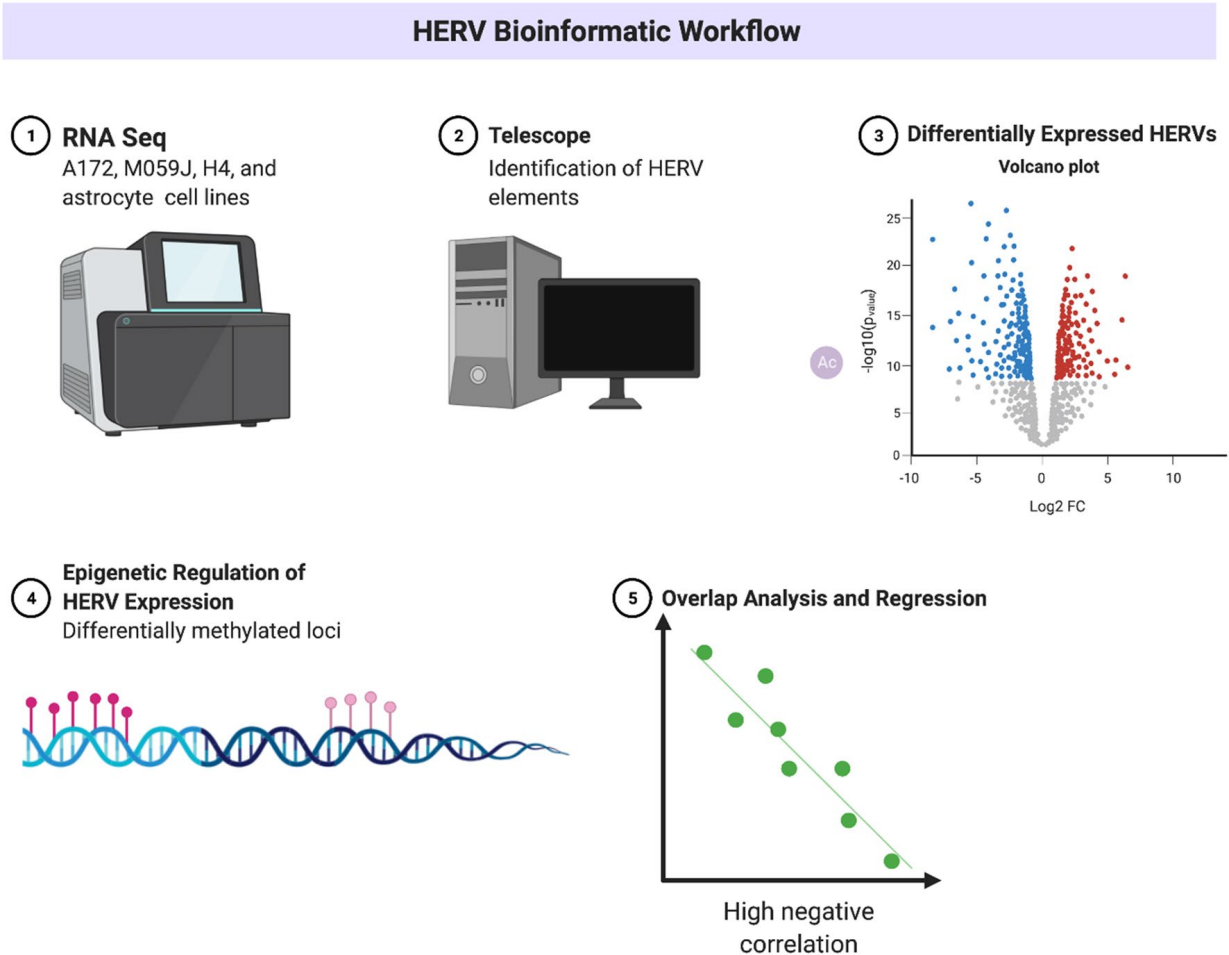
Summary of bioinformatics workflow. Created with BioRender.com.

To analyze methylation patterns in our cell lines, ‘.idat’ files from the ENCODE project were utilized. We created CSV files containing the basic metadata of our ‘.idat’ files and used the R packages minfi and illuminaio to read our ‘.idat’ files as methylation arrays. The resultant arrays were preprocessed to generate Methylation Sets (MSs) by normalizing the data, performing outlier assessment, and probe filtering. As in our HERV expression analysis, we employed quality control procedures to ensure that the analyzed methylation probes were ordered correctly and to remove any probes with SNPs at CpG islands. We also collected M (log2 fold ratio of methylated probes to unmethylated probes) and beta-values (percentage of methylated probes) before generating a model to assess methylation pattern by cell line. To assess differential methylation sites, we fit the model using Bayesian estimators and mapped the differentially methylated sites against the Illumina Human Methylation 450 K annotation. The locations of the differentially methylated loci were noted (corrected *p* < 0.05) and then compared against the positions of our HERVs of interest. Finally, we identified genes that overlapped our Differentially Methylated Regions (DMR). A summary of our overall workflow is shown in Fig. 6 .

### Cell culture

A172 and Normal Human Astrocytes (NHA) were obtained from the American Type Culture Collection (ATCC) and maintained in recommended culture conditions: A172 (Dulbecco’s Modified Eagle Medium (DMEM) with 5% fetal bovine serum (FBS) supplemented with penicillin/streptomycin) and NHA (Astrocyte Growth Medium Bullet Kit, Lonza). Non-adherent glioma patient-derived neurospheres (GBM28, 68-year old male with IDH wild-type gliosarcoma and GBM43, 69-year old male with IDH wild-type glioblastoma) were obtained from the Mayo Clinic Brain Tumor Patient Derived Xenograft National Resource^40^ and maintained in serum-free media, DMEM/F12 without phenol red (Invitrogen), supplemented with 20 ng/mL epidermal growth factor (EGF) and fibroblast growth factor (FGF), 2% B27 (Invitrogen), 1% penicillin–streptomycin and 1% sodium pyruvate (FisherScientific). TrypLE Express (Invitrogen) was used to dissociate neurospheres.

### Primer design

To validate our bioinformatics findings, we generated primers specific for the highest expressed HERV in our data set, Human endogenous Mouse mammary tumor (MMTV)-Like virus 6 (*HML-6*). The *HML-6* group is a member of the beta retrovirus superfamily (Class II) and includes several pro-viral loci and an intact open reading frame (ERVK3-1). The sequence for ERVK3-1 locus on Chromosome 19q13.43b was retrieved from dFam^20^ and University of California Santa Cruz Genome Browser using Repeat Masker^21^. Using primer3, two sets of primers for the *HML-6* envelope locus ERVK3-1 were established (https://primer3.ut.ee) and validated for target specificity for the chromosome 19 locus using UCSC in-silico PCR (hg38)^41–43^.

### RT-PCR and qPCR of HML-6 transcripts

RNA was isolated from NHA, A172, GBM28, and GBM43 using Trizol extraction with chloroform as previously described^44^. RNA was purified using DNase and quantified using NanoDrop 2000 (ThermoFisher Scientific) for a 260/280 value ~ 2.0, and reverse transcribed using the SuperScript First-Strand Synthesis RT-PCR kit (Thermo Fisher Scientific). Polymerase chain reactions were then used to amplify *HML-6* transcripts on the cDNA using primers (10 uM) which target the protein-coding region of *HML-6* (ERVK3-1). Fast SybR green master mix was used for the PCR with the following cycling conditions: 95 °C for 20 s, [95 °C for 3 s, 60 °C for 30 s] repeated for 40 cycles followed by 95 °C for 20 s [95 °C for 1 s, 60 °C for 20 s] for 40 cycles. Both primers targeted the env region of the HERVK3-1 downstream of LTR13. Ct values were normalized to expression of NHA using the delta-delta Ct method^45^. Reverse transcriptase negative controls were included and did not amplify.

### Sanger sequencing

To confirm amplicon sequence, our primers were validated using both in silico* PCR (*UCSC Genome Browser) and Sanger Sequencing. Briefly, PCR products for both primer sets for ERVK3-1 were amplified using Q5 High-Fidelity DNA polymerase (New England BioLabs, M0491S). Amplified product was run on a 1.8% agarose gel with GelStar Nucleic Acid Gel stain (Lonza, 50,535). A single DNA band was localized around 170–190 bp, excised and DNA was extracted using the QIAquick gel extraction kit (Qiagen, 28,704) according to manufacturer’s protocol. The extracted DNA was amplified using GoTaq Polymerase (Promega, M3001). The amplified product was cloned using the TOPO TA Cloning kit for Sequencing with One Shot TOP10 Chemically Competent *E. coli* (ThermoFisher Scientific, K203001). Transformants were analyzed, grown overnight in 5 mL of LB and purified using QIAprep Spin Miniprep kit (Qiagen, 12,843). Plasmid DNA was mixed with M13 Forward (− 20) Primer (5′-GTAAAACGACGGCCAG-3′) and sent for Sanger Sequencing to confirm the exact locus of HML-6 transcription in our samples. Sanger sequencing matched the PCR amplicon of ERVK3-1 for both primers. Results of sequencing are displayed in the Supplemental Material.

### RNA in situ hybridization

Visualization of RNA transcripts was performed using RNA-scope Multiplex Assay v2 (Advanced Cell Diagnostics, Hayward, CA) according to the manufacturer’s instructions. A172 cells were fixed using 4% paraformaldehyde on chambered slides, dehydrated, and rehydrated before probe hybridization. Probes specific for the *HML-6* locus at chromosome 19q13.43b were established per manufacturer’s recommendation. The C2 probe for *HML-6* was hybridized in the HybEZ Oven at 40 °C using Opal620 (red) fluorophores. Slides were co-stained with DAPI mounting media prior to microscopy. Bright-field images were acquired using a Leica SP8 confocal microscope (40X magnification, far red = 635 nm, green = 488 nm). All images were analyzed using Imaris (Bitplane version 9.3). The presence of red punctate dots was considered positive for *HML-6* transcripts. Negative controls were included per manufacturer protocol (ACD Bio). Sequence of the C2 probe is found in the Supplemental Material.

### Analysis of clinical GBM samples

Clinical information including ERVK3-1 expression was collected from The Cancer Genome Atlas (TCGA) and Human Protein Atlas (HPA) using the R package TCGABiolinks. Outliers were filtered using the R package survbootOutliers. We employed the OneStepDeletion (OSD) algorithm to maximize the concordance of the remaining data using a cox-regression model (c-index). Patient data was then further stratified by IDH mutation status and GBM subtype (classical, mesenchymal, and proneural) for further analysis. Comparisons by GBM subtype were analyzed by multiple comparisons ANOVA with Tukey’s correction. Subsequently, TCGA findings were validated using data downloaded from the CGGA database, downloaded via GlioVis.

## Supplementary Information

Supplementary Information.

## Data Availability

RNAseq data is publicly available via the ENCODE project (encodeproject.org), clinical sample data is publicly available in The Cancer Genome Atlas (TCGA, cancer.gov).
