# Supplementary material for: Differential expression of an endogenous retroviral element [HERV-K(HML-6)] is associated with reduced survival in glioblastoma patients
Source: Sci Rep. 2022 Apr 27;12:6902. doi: 10.1038/s41598-022-10914-5 (PMC9046263; doi:10.1038/s41598-022-10914-5)
Supplement: Supplementary file 1 — Supplementary Information. [file 41598_2022_10914_MOESM1_ESM.pdf]

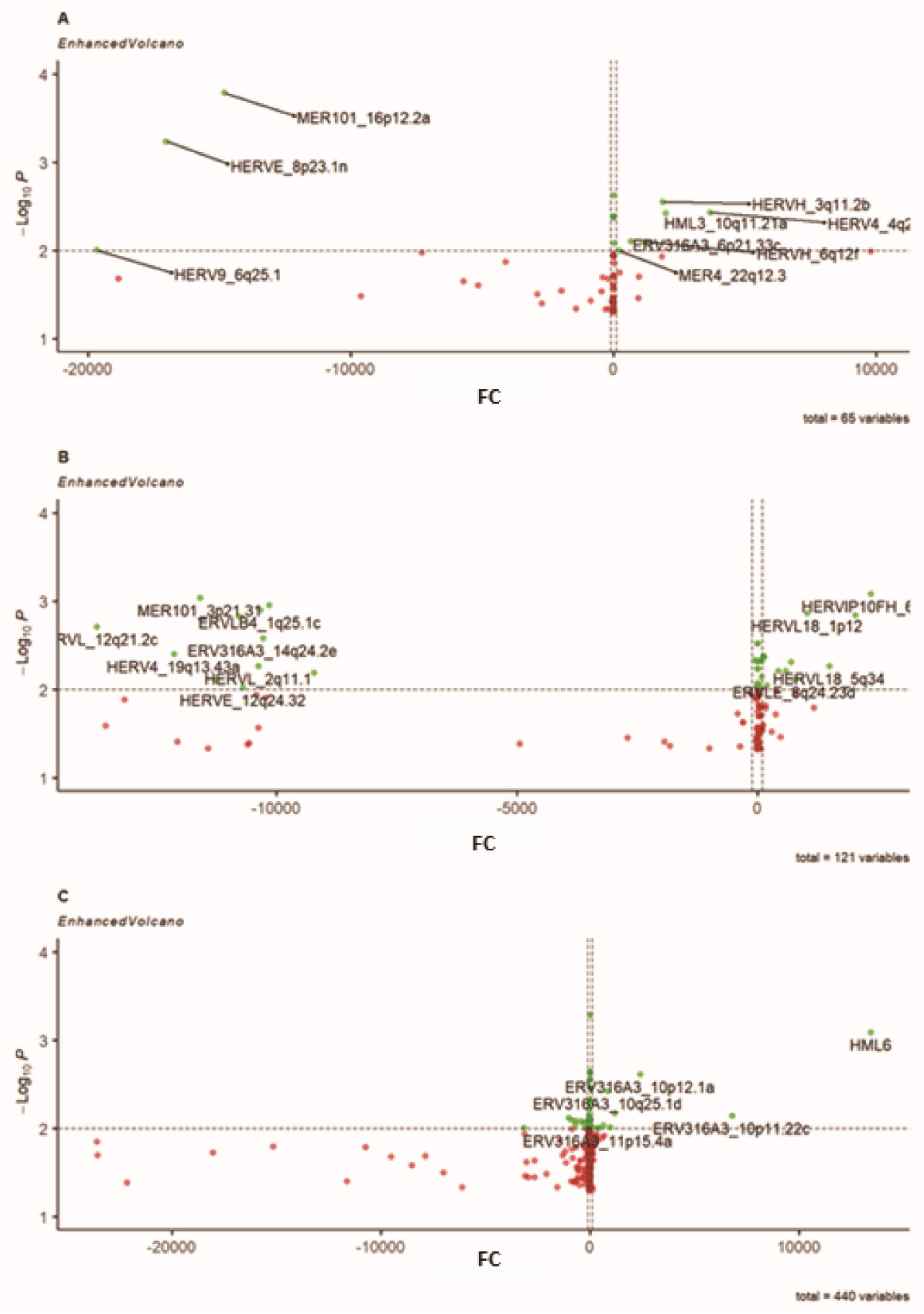

Supplemental Figure 1. Summary of differential HERV expression in M059J (A), H4 (B), and A172 (C) cell lines.

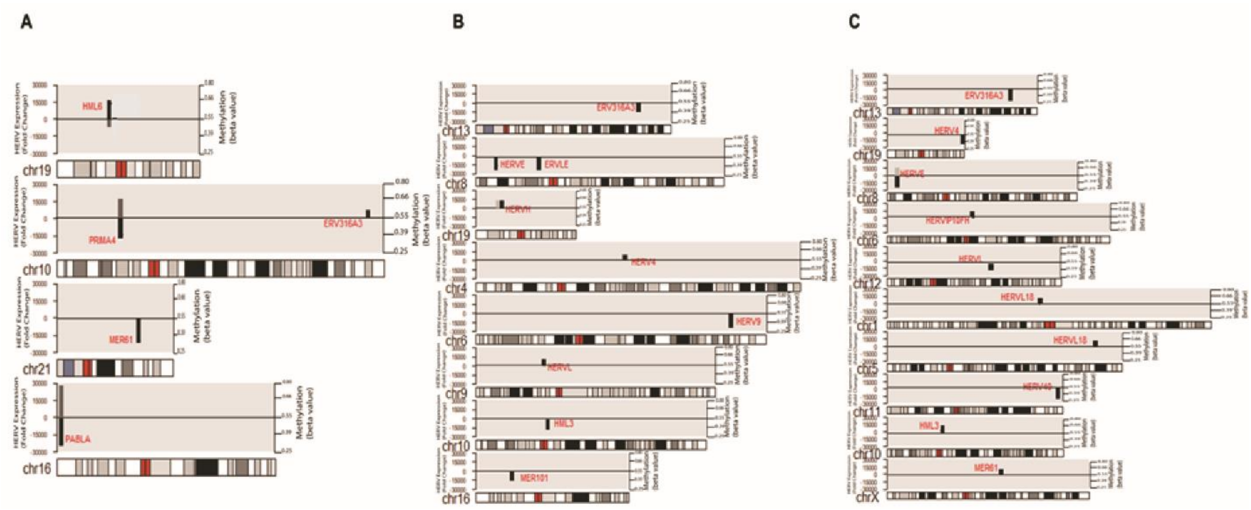

Supplemental Figure 2. Summary of differentially methylated regions containing HERVs

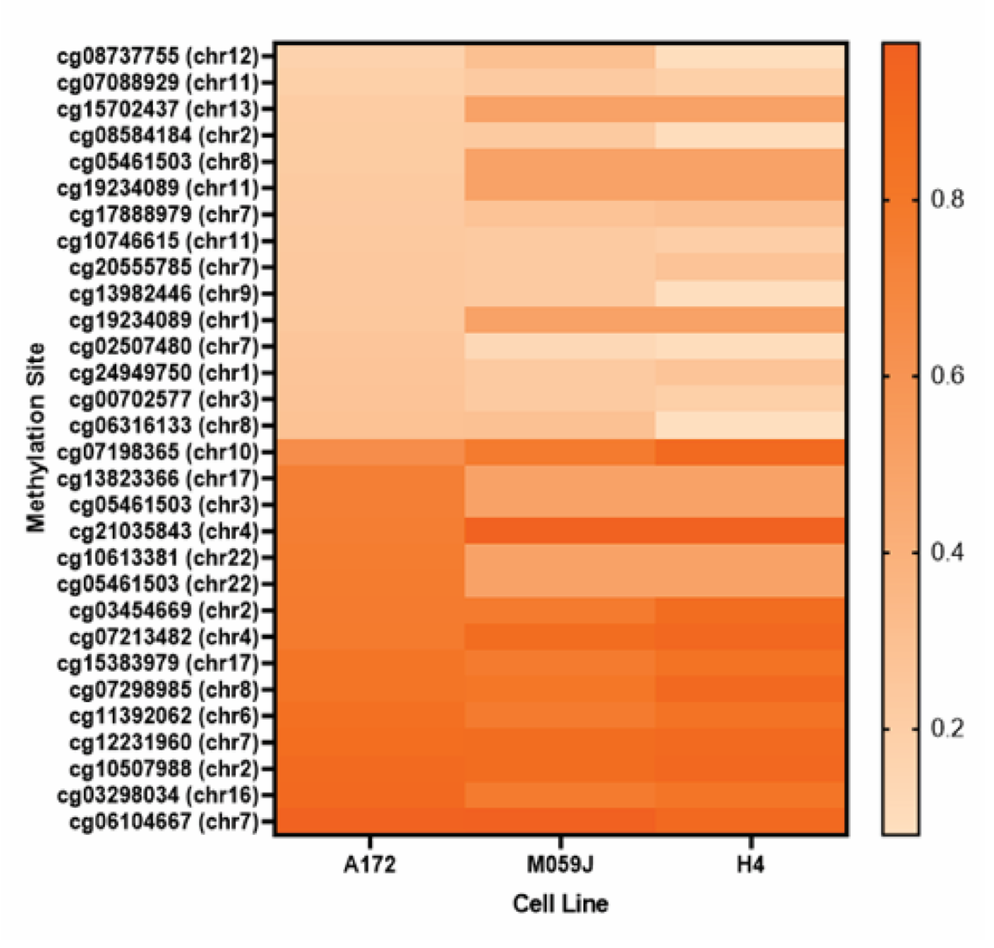

Supplementary Figure 3. Summary of methylation (beta value) by most differentially methylated regions of each investigated cell line.

HERV	Locus	FC HERV	Differentially Methylated Gene	FC Gene	Methylation	Gene Function	Potential Anti-sense RNA
**PRIMA4**	10p12.1	-14893	PDSS1	-1.35	0.63	Key respiratory chain element	Yes
**PABLA**	16p13.3	-22070	MLST8	1.34	0.74	mTOR-associated protein	Yes
**HML6**	19q13.43b	13162	ZNF208	1.10 (NS)	0.45	Zinc finger	Yes
**PABLA_Xp22.2**	Xp22.2	-2537.73	CTPS2	1.22	0.46	Rate limiting enzyme in production of cytosine nucleotides	No
**ERV316A3_11q24.2**	11q24.2	181.43	DDX25	2.74	0.57	RNA helicase involved in several functions: translation initiation, nuclear and mitochondrial splicing, and ribosome and spliceosome assembly. May also involve cellular growth and division	Yes
**HARLEQUIN_7q22.1**	7q22.1	-871.225	DGAT2L7P	1.32	0.56	Pseudogene associated with metabolism	No

Supplemental Table 1. Summary of DEG-DEHERV pairs at differentially methylated sites along with identification of potential antisense RNA sequences in A172 and M059

Sequenced PCR Product at 19q13.43b

tgttaccTCCAGTACGAGCCATGAGCCAGCGGAATCTGAATGCAAAGACAGAACAAGGG
CCGACCGGAGTCACAATGACATCCAACCCCATAACATGGGGACAGATCAAGAAAAC
GACACAAGAAGCTGAGAAACTACTGGAGCGCCAGGGTCAGGCAAAAACCCCTGACT
CCAtgttct

Alignment of YourSeq and chr19:58312254-58312433

Click on links in the frame to the left to navigate through the alignment. Matching bases in cDNA and genomic sequences are colored blue and capitalized. Light blue bases mark the boundaries of gaps in either sequence (often splice sites).

cDNA YourSeq

```
cacgactgaa taaccctcac taaagggact agtcctgcag gtttaaacga  50
attcgccctT GTTACCTCCA GTACGAGCCA TGAGCCAGCG GAATCTGAAT  100
GCAAAGACAG AACAAGGGCC GACCGGAGTC ACAATGACAT CCAACCCCAT  150
AACATGGGGA CAGATCAAGA AAACGACACA AGAAGCTGAG AAACTACTGG  200
AGCGCCAGGG TCAGGCAAAA ACCCCTGACT CCATGTTCTa agggcgaatt  250
cgcggccgct aaattcaatt cgccctatag tgagtcgtat tacaattcac  300
tggccgtcgt tttacaacgt cgtgactggg aaaaccctgg cgttacccaa  350
cttaatcgcc ttgcagcaca tccccctttc gccagctggc gtaatagcga  400
agaggcccgc accgatcgcc cttcccaaca gttgcgcagc ctatacgtac  450
ggcagtttaa ggtttacacc tataaaagag agagccgtta tcgtctgttt  500
gtggatgtac agagtgatat tattgacacg ccggggcgac ggatggtgat  550
ccccctggcc agtgcacgtc tgctgtcaga taaagtctcc cgtgaacttt  600
acccggtggt gcatatcggg gatgaaagct ggcgcatgat gaccaccgat  650
atggccagtg tgccggtctc cgttatcggg gaagaagtgg ctgatctcag  700
ccaccgcgaa aatgacatca aaaacgccat taacctgatg ttctggggaa  750
tataaatgtc aggcatgaga ttatcaaaaa ggatcttcac ctagatcctt  800
ttcacgtaga aagccagtcc gcagaaacgg tgctgacccc ggatgaatgt  850
cagctactgg gctatctgga caagggaaaa cgcaagcgca aagagaaagc  900
aggtagcttg cagtgggctt acatggcgat agctagactg ggcggtttta  950
tggacagcaa gcgaaccgga attgccagct ggggcgccct ctggtaaggt  1000
tgggaagccc tgcaaagtaa actggatggc tttcttgccg ccaaggatct  1050
ganggcccag gggatcaatn tttgatc
```

Genomic chr19 :

```
gtgtttttac aggagatgga caaaccgtgt gggtgccctc caggtgtgtg  58312203
tgaccatgga acgggagacc ggagggatcc atggtattca accgtgggcc  58312253
TGTTACCTCC AGTACGAGCC ATGAGCCAGC GGAATCTGAA TGCAAAGACA  58312303
GAACAAGGGC CGACCGGAGT CACAATGACA TCCAACCCCA TAACATGGGG  58312353
ACAGATCAAG AAAACGACAC AAGAAGCTGA GAAACTACTG GAGCGCCAGG  58312403
GTCAGGCAAA AACCCCTGAC TCCATGTTCT tggccatgct agctgtagtg  58312453
tcctgtgcgg tatgtttccc ctgtgtagag gcaaaaacat attgggcata  58312503
tgttcctaac ccactggtag tacgactggt
```

Sequence of C2 Probe

NR_144445.1 ERVK3-1

C2 probe

2 gcagtgtgt aagacgctca cgggcgcgcg gactatcggg cggctaggct ctctgaggag

61 gctgccacag tgaagcaacc gtgacaagtg gtgcccgacc agggacctga acgaggaagg

121 tctgccagag cagagaaagt gaaactgatc agacgaacta cgaacccctg gacgggagag

181 tctgccggcg gagaatataa ggagatggac aaaccgtgtg ggtgccctcc aggtgtgtgt

241 gaccatggaa cgggagaccg gagggatcca tggtattcaa ccgtgggcct gttacctcca

301 gtacgagcca tgagccagcg gaatctgaat gcaaagacag aacaagggcc gaccggagtc

361 acaatgacat ccaaccccat aacatgggga cagatcaaga aaacgacaca agaagctgag

421 aaactactgg agcgccaggg tcaggcaaaa acccctgact ccatgttctt ggccatgcta

481 gctgtagtgt cctgtgcgtc tataggatcg ggtgaaccac caaccggcaa ttgaaagctg

541 cacagcctga aattaccttt attcaattaa tgcaaaaaca aaaggggaga tgttggaggc

601 cgaaggaatg agggtcgtga ccaactcagt ataccactgg aggctctgtg agcaaacagc

661 aaactgttct catgaaagca ggatgttggc gaactgacaa actctgtctg ccgccagaag

721 gaatgctggg ggcagtcatg tcccaggtcc cgtgctcctt gaggttatcc acaggaacat

781 ccagaacctg ttgaacaaag aaaggaatca tgtgggcctg tgttaaatca aatagctgac

841 cgacagttat cccttcctcc ctattcactc tacctaataa atatgaaggg ctgtaaaagc

901 tcaggtccct gttccctaat atcaaggagc cccctgaccc cttctttcaa acagatcctt

961 ttgtctgtct catttctgca ttcgtcgtcc ttcgttcggt ccagaagcaa ccgcgacagt

1021 cctgcatgtg ttgatgccac ctgtatgtgc aggtgtgacc tcaggtgtgt gagtatccct

1081 catacccctt atcatcactg cctattcgct cttcctccag cctctggcaa ccactaatcc

1141 acgttatatg tctagat
